# Attribution of antibacterial and antioxidant activity of *Cassia tora* extract toward its growth promoting effect in broiler birds

**DOI:** 10.14202/vetworld.2017.221-226

**Published:** 2017-02-19

**Authors:** Jyoti Sahu, K. M. Koley, B. D. Sahu

**Affiliations:** 1 Department of Veterinary Pharmacology and Toxicology, College of Veterinary Science and Animal Husbandry, Anjora, Durg - 491 001, Chhattisgarh, India; 2 Department of Livestock Development, Dondi, Balod - 491 226, Chhattisgarh, India

**Keywords:** antibacterial activity, antioxidant activity, broiler birds, *Cassia tora*, growth promoter

## Abstract

**Aim:**

The study was conducted to evaluate the attribution of antibacterial and antioxidant activity of methanolic extract of *Cassia tora* toward its growth promoting effect in broiler birds.

**Materials and Methods:**

A limit test was conducted for *C. tora* extract in Wistar albino rats. Phytochemical screening of methanolic extract of leaves of *C. tora* was carried out. In-vitro antibacterial activity was measured by disc diffusion method. 1-day-old Ven Cobb broiler birds (n=90) were randomly allocated into three groups consisting of three replicates with 10 birds in each group. The birds of group T1 (Control) received basal diet, whereas birds of group T2 (Standard) received an antibiotic (Lincomycin at 0.05% in feed). The birds of group T3 (Test) received *Cassia tora* extract (CSE) at 0.4 g/L in drinking water in addition to basal diet. The treatment was given to birds of all the groups for 6 weeks. Antioxidant activity of *C. tora* was determined in blood of broiler birds. Cumulative body weight gain, feed intake, feed conversion ratio (FCR), dressing percent, and organ weight factor were evaluated to determine growth performance in broiler birds.

**Results:**

Phytochemicals in *C. tora* were screened. Sensitivity to *Escherichia coli* and resistant to *Staphylococcus aureus* and *Pseudomonas aeruginosa* was observed in *in-vitro* antibacterial activity test. At the end of 6^th^ week, antioxidant activity reflected significantly (p≤0.05) lower level of erythrocyte malondialdehyde and higher levels of reduced glutathione (GSH) and GSH peroxidase in broiler birds of group T2 and T3 as compared to broiler of group T1. Mean cumulative body weight gain of birds of T2 and T3 were significantly (p≤0.05) higher as compared to T1. Mean FCR of birds of group T3 decreased significantly than group T1.

**Conclusion:**

Supplementation of *C. tora* leaves extract at 0.4 g/L in drinking water improved growth performance in broiler birds due to its antimicrobial and antioxidant activity. Therefore, it could be used as an alternative to antibiotic growth promoter in poultry ration.

## Introduction

During the last four decades, Poultry Industry in India has transformed itself from the age-old backyard farming into a dynamic agri-based industry. India’s population is about 1096.9 million in 2005 and with the growth rate of 1.6% per year another 450 million people will be added to the existing population by 2025. The current per-capita consumption (availability) of eggs is 49 while chicken meat consumption is 1.9 kg. It is far below the recommended consumption of 180 eggs and 10.8 kg poultry meat per person per annum. Thus to meet the nutritional requirement, the layer and broiler industry has to grow 5- and 10-folds, respectively [1].

Subtherapeutic feeding of antibiotics has historically been a practice in various sectors of the commercial broiler industry to promote growth performance and protect flock health. However, the overuse and misuse of antibiotics leads to the development of drug-resistant bacteria, and the drug residues in the meat also threaten consumer health. Consequently, the drugs used become less effective, and some consumers have started to avoid certain meat products. In January 2006 the European Union (EC Regulation No. 1831/2003) approved a resolution to ban the use of antibiotics as growth promoter in animals [2]. Many natural compounds used as alternatives to antibiotics in animal feed were shown to express positive effects on growth performance and health parameters. Therefore, there is an urgent need to investigate alternative growth promoter which gives similar results in improving poultry performance.

Since ancient times *Cassia tora* (Common name: Charota/Chakunda) has been a subject of considerable interest as herbal medicine worldwide. *C. tora*, plant of *Caesalpiniaceae* family, is an important legume crop in Indian subcontinent, Eastern Africa, and Central America. The leaves and seeds of *C. tora* are reported to have curative effect in leprosy, flatulence, colic, dyspepsia, constipation, cough, bronchitis, cardiac disorders, skin diseases, and liver disorders [3]. *C. tora* leaves were found positive for phenols, tannins, saponins, glycosides, flavonoids, steroids, and alkaloids [4]. In the backdrop of facts, the study was conducted to determine the antibacterial and antioxidant activity of methanolic extract of *C. tora* leaves (CSE) in suitable study model thereby investigating its role as an alternative to antibiotic growth promoter in broiler birds.

## Materials and Methods

### Ethical approval

The study was conducted in the Department of Veterinary Pharmacology and Toxicology, College of Veterinary Science and Animal Husbandry, Anjora, Durg, Chhattisgarh, India. All the animals and procedure employed were approved by the Institutional Animals Ethics Committee as well as Committee for the Purpose of Control and Supervision of Experiments on Animals.

### Plant extract

The leaves of *C. tora* used in the study were collected locally, identified, shed dried, and powdered using an electric grinder. The methanolic extract from the powder was prepared by using Soxhlet’s apparatus by hot extraction technique. The obtained extract was evaporated on water bath to give dried residues. The dried extract was labeled and stored in airtight screw cap box in refrigerator at 4°C until use.

### Limit test

Acute oral toxicity study (Limit test) was performed as per OECD guidelines [5] for testing of CSE in Wistar albino rats with upper limit dose of 2000 mg/kg b.wt. The mortality, behavioral abnormality, signs and symptoms of toxicity, if any, were recorded for 14 days post administration.

### Phyotochemical screening

The freshly prepared methanolic extracts of *C. tora* were subjected to preliminary phytochemical screening for the presence or absence of various active metabolites using standard chemical tests as per method described by Raaman [6].

### Antibacterial activity test

The antibacterial activity was investigated against pure cultures of pathogenic strains of *Escherichia coli, Staphylococcus aureus, Streptococcus pyogenes, Salmonella gallinarum*, and *Pseudomonas aeruginosa* by disc diffusion technique [7]. Bacteria were subcultured on nutrient broth at 37°C overnight. 1 ml of the broth culture of each bacterium was spread over the nutrient agar taken in Petri dishes aseptically. The filter paper discs (6 mm diameter) saturated with a solution of CSE (concentrations 100 and 200 mg/ml in distilled water) and a reference antibiotic disc (ciprofloxacin 5 µg/disc) were placed over it and incubated at 37°C for 24 h. The Petri-dishes were observed for the presence of zone of inhibition around the discs and were measured by zone reader scale in millimeter. The tests were repeated thrice to confirm the findings, and the average of the readings was taken into consideration.

### Experimental design for study on antioxidant and growth parameters

1-day-old birds (n=90) were randomly allocated into three groups consisting of three replicates with 10 birds in each replicate. All the groups were maintained as per the following treatment schedule for 6 weeks: Group T1 (Control): Basal diet, Group T2 (Standard): Basal diet supplemented with Lincomycin at 0.05% w/w in feed and Group T3 (Test): CSE at 0.4 g/L w/v in drinking water in addition to basal diet. Composition of broiler starter, grower, and finisher diet has been presented in Table-1. All the birds were provided with feed and water *ad libitum* throughout the experiment period (6 weeks).

**Table-1:** Composition of broiler starter, grower and finisher diet (on % DM basis).

Ingredient	Starter	Grower	Finisher
Yellow maize	54.80	55.00	55.32
Deoiled soybean meal	37.00	33.40	28.47
Rice polish	2.60	7.00	10.00
Soybean oil	2.00	2.00	2.50
DCP	1.60	1.60	1.60
LSP	0.70	0.70	0.70
L-methionine	0.28	0.26	0.24
Lysine	0.04	0.02	0.17
Sodium bi carbonate	0.14	0.15	0.16
Common salt	0.28	0.29	0.26
Mineral mixture	0.56	0.58	0.58
Total	100.00	100.00	100.00
CP (%)	23.05	21.50	20.00
ME (kcal/kg)	2975.6	3017.0	3084.0

DCP=Dicalcium phosphate, LSP=Limestone powder, CP=Crude protein, ME=Metabolizable energy

### Antioxidant activity test

At the end of 6 weeks of the experiment, antioxidant status of birds was measured in blood. Lipid peroxidation (LPO) in red blood cell (RBC) hemolysate [8], reduced glutathione (GSH) concentration in RBC suspension [9], and GSH peroxidase (GPx) activity in RBC hemolysate [10] were determined as per standard method.

### Growth performance study

The body weight of bird and feed intake were recorded every week up to 6^th^ week of age. The weekly weight gain and feed conversion ratio (FCR) were calculated. At the end of the 6^th^ week study period, 6 birds of each group were slaughtered and examined to determine dressing percent and organ weight of empty gizzard, liver, and heart of each group to calculate the organ weight factor as per the following formula:


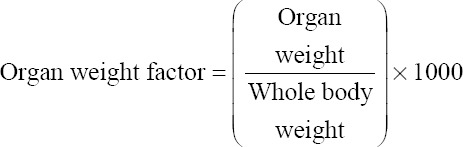


Then, sensory parameters (color, flavor, juiciness, tenderness, texture, and overall liking) by a panel of 6 semi-trained judges were recorded. Breast meat was used for sensory quality test according to the 9-point Hedonic scale [11].

### Statistical analysis

Data obtained were expressed as mean±standard error. The data were analyzed for statistical significance by one-way analysis of variance test using the Statistical Package of the Social Science (SPSS) version 16.0 (SPSS Inc., Chicago, IL, USA). Duncan’s multiple range test was applied to determine a significant difference (p≤0.05) if any, among the treatment groups.

## Results

### Limit test

Neither mortality nor the behavioral abnormality in the form of signs and symptoms of toxicity was recorded in limit test of CSE in rats. Therefore, oral lethal dose 50% of the CSE in rats was recorded above 2000 mg/kg b.wt.

### Phyotochemical screening

Soxhlet’s extraction of powder of *C. tora* yielded 4% of methanolic extract. Preliminary phytochemical screening of methanolic extract of *C. tora* revealed the presence of alkaloids, glycosides, flavonoids, phenols, phytosterols, saponins, tannins, and reducing sugar.

### Antibacterial activity test

Methanolic extract of *C. tora* (8.792 mg) showed antibacterial activity (Table-2) against all tested bacteria, but maximum activity was recorded against *E. coli* (25.20±0.49 mm) followed by *S. gallinarum* (18.60±0.24 mm), *S. aureus* (11.2±0.49 mm) whereas least activity against *P. aeruginosa* (7.20±0.37 mm) and *S. pyogenes* (7.20±0.37 mm).

**Table-2:** Antibacterial activity test of CSE taking ciprofloxacin as standard drug.

Component	Weight of CSE/drug in each disc	Zone of inhibition (mm±SE) against bacterial culture				

		*S. aureus*	*S. pyogenes*	*S. gallinarum*	*P. aeruginosa*	*E. coli*
CSE	8.792 mg	11.2±0.49	7.20±0.37	18.60±0.24	7.20±0.37	25.20±0.49
Ciprofloxacin	5 µg	18.20±0.37	15.2±0.58	25.6±0.40	15.2±0.37	34.2±0.58

*S. aureus*=*Staphylococcus aureus*, *S. pyogenes*=*Streptococcus pyogenes*, *S. gallinarum*=*Salmonella gallinarum*, *P. aeruginosa*=*Pseudomonas aeruginosa*, *E. coli*=*Escherichia coli*, SE=Standard error, CSE=Extract of leaves of *Cassia tora*

### Antioxidant activity test

The effect of supplementing *C. tora* leaves extract at 0.4 g/L in antioxidant status of broiler birds has been presented in Table-3. The birds of CSE treated group T3 (8.76±1.24 nmole/ml) showed significantly (p≤0.05) lowered malondialdehyde (MDA) level (lipid peroxidation) as compared to control group T1 (11.42±1.31). The mean reduced GSH level of *Cassia* treated group T3 (0.24±0.02 mole/g Hb) was recorded significantly (p≤0.05) higher than lincomycin treated group T2 (0.19±0.01 mole/g Hb) and control group T1 (0.14±0.02 mole/g Hb). Birds of CSE treated group T3 (219.80±26.6 units/ml) had a higher level of GPx than control group T1 (146.32±19.65 units/ml).

**Table-3:** Effect of supplementation of CSE on antioxidant activity in broiler birds.

Parameter (unit)	Group		

	Control (T1)	Standard (T2)	Test (T3)
LPO (nmole/ml)	11.42±1.31^a^	10.20±1.30^b^	8.76±1.24^c^
GSH (mole/g Hb)	0.14±0.02^c^	0.19±0.01^b^	0.24±0.02^a^
GPX (units/ml)	146.32±19.65^b^	159.93±27.13^b^	219.80±26.6^a^

Mean values with dissimilar superscripts within row vary significantly (p≤0.05). LPO=Lipid peroxidation, GSH=Glutathione, GPX=Glutathione peroxidase, Hb=Hemoglobin, CSE=Extract of leaves of *Cassia tora*

### Growth performance study

The effect of supplementing *C. tora* leaves extract at 0.4 g/l in growth performance of broiler birds has been presented in Table-4. The broiler birds of Cassia treated group T3 (1718.67±43.01 g) and lincomycin treated group T2 (1698.60±48.57 g) had significantly (p≤0.05) improved cumulative body weight gain at 6^th^ week by 9.14% and 7.87% above the birds of control group T1 (1574.67±23.97 g). At the 6^th^ week, the cumulative feed consumption of control, standard and test group were recorded 3250.70±41.11 g/bird, 3277.67±59.91 g/bird and 3347.23±59.46 g/bird, respectively (Table-4). The inclusion of 0.4 g/L CSE in drinking water significantly (p≤0.05) lowered cumulative FCR in birds of CSE treated group (1.95±0.01) than birds of control group (2.06±0.02) at the end of 6^th^ week of study.

**Table-4:** The effect of supplementing CSE at 0.4 g/L in growth performance in broiler birds.

Parameter (unit)	Group		

	Control (T1)	Standard (T2)	Test (T3)
Initial body weight (g)	36.93±2.32	36.87±1.56	36.47±3.67
Final body weight (g)	1611.60±44.20^b^	1735.47±38.40^a^	1755.13±42.52^a^
Cumulative body weight gain (g)	1574.67±23.97^b^	1698.60±48.57^a^	1718.67±43.01^a^
Total feed intake (g/bird)	3250.70±41.11	3277.67±59.91	3347.23±59.46
FCR	2.06±0.02^a^	1.93±0.03^b^	1.95±0.01^b^
Dressing %	71.48±4.21	74.63±3.21	75.25±6.08

Mean values with dissimilar superscripts within row vary significantly (p≤0.05). FCR=Feed conversion ratio, CSE=Extract of leaves of *Cassia tora*

The dressing percent was higher in test group (75.25 ± 6.08%) as compared to control group (71.48 ± 4.21%). As shown in Table-5, the organ weight factor of empty gizzard of control group (22.42±5.65) and CSE treated group (22.44±1.46) increased significantly (p≤0.05) when compared to standard group (20.77±5.65). The organ weight factor for liver varied non-significantly (p≤0.05) among the birds of control, standard and test groups (T1, T2 and T3). The organ weight factor for heart was recorded higher non-significantly (p≤0.05) in broiler birds of *Cassia* treated group (5.19±0.24) than standard (4.50±0.56) and control group (4.88±0.24).

**Table-5:** Effect of supplementing CSE on organ weight factor in broiler birds (n=6).

Organ weight factor	Group			

	Control (T1)	Standard (T2)	Test (T3)
Empty gizzard	22.42±5.65^a^	20.77±5.65^b^	22.44±1.46^a^
Liver	22.90±1.28	23.07±0.55	23.32±0.49
Heart	4.88±0.24	4.50±0.56	5.19±0.24

Mean values with dissimilar superscripts within row vary significantly (p≤0.05). CSE=Extract of leaves of *Cassia tora*

## Discussion

Many experimental studies have suggested that plant extracts have increased gut microflora, which positively affects host nutrition, health and growth by better utilization of nutrients. There is evidence that plant contents have digestion stimulating properties that have intrinsic bioactivities on animal physiology and metabolism [12]. Studies have shown that the active agents in the herbs have a strong capability for scavenging superoxide radicals, hydrogen peroxide and nitric oxide from activated macrophages, reducing iron complex and inhibiting lipid peroxidation. Apart from the digestive and antioxidant prosperities, the herbs and plant additives may exert the beneficial influence through anti-microbial, immuno-modulating and antiparasitic effects [13].

The present outcome corroborated with the findings of Shaikh and Syed (2015) who reported that the *C. tora* leaves were positive for phenols, tannins, saponins, glycosides, flavonoids, steroids and alkaloids in addition to magnesium, calcium, sulfur, iron, sodium and chlorine [4]. The flavonoids (phytochemical) exhibit the beneficial effect due to the antioxidants and chelating properties of these molecules by preventing the free radicals to damage the biological molecules such as lipid, protein, sugar, DNA, and RNA. The free radicals are having at least one free electron in their outer orbit and produced from ionization of oxygen such as reactive oxygen species superoxide anion, hydroxyl, and peroxyl radicals. These radicals cause cell death by attacking on deoxyribose and nitrogen bases, breaking the DNA strands and accumulation of mutations. This damage the proteins and disulfide bonds or by nitration of aromatic amino acids. Utilization of the plant extracts as an alternative to chemical or synthetic antimicrobials and antioxidants to control the foodborne diseases, inhibiting lipid oxidation and thus extending the shelf life and quality of food products is an increasing trend in the food industry [14]. The mechanisms of flavonoids that are known for its antimicrobial activity involved inhibition of nucleic acid synthesis, cytoplasmic membrane function, and energy metabolism [15].

*In-vitro* studies on antimicrobial activity of methanolic extract of *C. tora* illustrated its sensitivity to *E. coli*, Gram-negative bacteria, which is a major culprit in broiler production. Lesser antimicrobial activity was shown by CSE against *S. gallinarum* and almost resistant to *S. aureus, S. pyogenes*, and *P. aeuroginosa*. The flavonoids and steroids present in *C. tora* extracts have been reported to possess antimicrobial property [4]. Many mechanisms of antimicrobial actions of phytochemicals have been suggested by different researchers. Phytochemicals may exhibit different modes of action against enterotoxigenic bacterial strains which range from interference with the phospholipoidal cell membranes, which has as a consequence of increasing the permeability profile and loss of cellular constituents, damage of the enzymes involved in the production of cellular energy and synthesis of structural components, and destruction or inactivation of genetic material [16]. The antimicrobial activities of emodin isolated from *C. tora* and its derivatives (anthraquinone, alizarin, and alizarin-3-methyliminodiacetic acid) against foodborne bacteria (*Bacillus cereus, Listeria monocytogenes, Staphylococcus intermedius, Salmonella typhimurium*, and *Shigella sonnei*) were described [17].

The antioxidant activity was studied in RBC of broiler bird supplemented with methanolic extract of *C. tora* at 0.4 g/L in drinking water. The significant decrease in LPO and increase in GSH and GPx in blood of CSE treated broiler birds than control broiler birds might be due to the presence of phenolic compounds, flavonoids and tannis [4,16]. Non-significant decrease in MDA and catalase levels of broiler on feeding 5-10% protein of *Azolla* with enzyme have been recorded which might be due to the presence of vitamins, iron, and copper, respectively [18]. These compounds are contributing in antioxidant activity of CSE. The antioxidant activity and the inhibition of free radical generation are important in terms of protecting the liver from damage. GPx is an important marker in protecting the liver from damage of lipid peroxidation. GPx is GSH related enzymes, which can catalyze the synthesis of GSH-S-transferase to ease the burden of lipid peroxidation [19,20]. Plant contains a wide variety of free radicals scavenging molecules including phenols, flavonoids, vitamins, terpenoids that are rich in antioxidant activity. Many dietary polyphenolic constituents derived from plants are more effective antioxidants *in vitro* than vitamins E or C, and thus might contribute significantly to protective effects *in vivo* [16]. The potent *in-vitro* antioxidant activity in methanolic extract of the leaves of *C. tora* was determined was attributed to the phenolics present in *C. tora* [19].

Significant improvement in the growth performance in broiler birds of CSE treated group may be due to the performance enhancing and antistress activity of *C. tora*. The cumulative body weight gain has increased and FCR decreased significantly in CSE-treated birds than birds of the control group. Similarly, Kirubakaran *et al*. [21] observed significantly higher cumulative weight gain, feed consumption and lower FCR in broilers fed with fenugreek, garlic and black pepper combinations than control broilers at 6 weeks of age. The body weight gain of birds could be attributed to flavonoids compounds. Flavonoids have been reported to possess many useful properties, containing anti-inflammatory activity, antimicrobial activity, estrogenic activity, anti-allergic activity, antioxidant activity [4]. The dressing percent and sensory evaluation improved in *C. tora* treated broiler birds than nontreated birds. Similarly, 5% *C. tora* leaves meal inclusion in chicken has significantly improved the body weight and the mean carcass weight when compared to the control group [22]. Empty gizzard, liver and heart are the edible portion of broiler meat. The organ weight factor of this portion was compared among control, standard and *Cassia* treated groups. Similarly, the weights of empty gizzard, liver, and heart were evaluated to study the effect of feeding probiotic and prebiotic on carcass characteristics and economics of commercial broilers [23]. Among all the groups, the broiler birds of CSE treated group scored significantly (p≤0.05) higher scores for juiciness, tenderness and overall acceptance as compared to control and standard group.

## Conclusion

Antimicrobial and antioxidant activity of *C. tora*, in association with many other medicinal properties such as hepatoprotective, antigenotoxic, purgative, hypolipidemic, and anthelmintic activities might be attributing toward growth promoting effect of *C. tora* in broiler birds. Thus, the supplementation of *C. tora* extract at 0.4 g/L in drinking water could be employed as an alternative to antibiotic growth promoter in broiler birds.

## Authors’ Contributions

KMK designed the experiment. JS carried out the research and recorded the data. BDS analyzed the data. JS, KMK, and BDS drafted and revised the manuscript. All authors have read and approved the final manuscript.
